# Literature Mining for the Discovery of Hidden Connections between Drugs, Genes and Diseases

**DOI:** 10.1371/journal.pcbi.1000943

**Published:** 2010-09-23

**Authors:** Raoul Frijters, Marianne van Vugt, Ruben Smeets, René van Schaik, Jacob de Vlieg, Wynand Alkema

**Affiliations:** 1 Computational Drug Discovery (CDD), Nijmegen Centre for Molecular Life Sciences (NCMLS), Radboud University Nijmegen Medical Centre, Nijmegen, The Netherlands; 2 Department of Immune Therapeutics, Schering-Plough, Oss, The Netherlands; 3 Department of Molecular Design & Informatics, Schering-Plough, Oss, The Netherlands; University of Chicago, United States of America

## Abstract

The scientific literature represents a rich source for retrieval of knowledge on associations between biomedical concepts such as genes, diseases and cellular processes. A commonly used method to establish relationships between biomedical concepts from literature is co-occurrence. Apart from its use in knowledge retrieval, the co-occurrence method is also well-suited to discover new, hidden relationships between biomedical concepts following a simple ABC-principle, in which A and C have no direct relationship, but are connected via shared B-intermediates. In this paper we describe CoPub Discovery, a tool that mines the literature for new relationships between biomedical concepts. Statistical analysis using ROC curves showed that CoPub Discovery performed well over a wide range of settings and keyword thesauri. We subsequently used CoPub Discovery to search for new relationships between genes, drugs, pathways and diseases. Several of the newly found relationships were validated using independent literature sources. In addition, new predicted relationships between compounds and cell proliferation were validated and confirmed experimentally in an *in vitro* cell proliferation assay. The results show that CoPub Discovery is able to identify novel associations between genes, drugs, pathways and diseases that have a high probability of being biologically valid. This makes CoPub Discovery a useful tool to unravel the mechanisms behind disease, to find novel drug targets, or to find novel applications for existing drugs.

## Author Summary

The biomedical literature is an important source of knowledge on the function of genes and on the mechanisms by which these genes regulate cellular processes. Several text mining approaches have been developed to leverage this rich source of information by automatically extracting associations between concepts such as genes, diseases and drugs from a large body of text. Here, we describe a new method that extracts novel, not yet recognized associations between genes, diseases, drugs and cellular processes from the biomedical literature. Our method is built on the assumption that even if two concepts do not have a direct connection in literature, they may be functionally related if they are both connected to an overlapping set of concepts. Using this approach we predicted several novel connections between genes, diseases, drugs and pathways. Our results imply that our method is able to predict novel relationships from literature and, most importantly, that these newly identified relationships are biologically relevant. Our method can aid the drug discovery process where it can be used to find novel drug targets, increase insight in mode of action of a drug or find novel applications for known drugs.

## Introduction

A wealth of knowledge concerning the function of genes and their role in biological processes is present in the biomedical literature, embodied in full text articles or the Medline abstract database. Various text mining approaches have been developed to extract information on gene function from this body of literature [1], [2] and these have been successfully applied to annotate genes and proteins [3]–[7] and the interpretation of experimental results [8]–[14].

**Figure 1:**
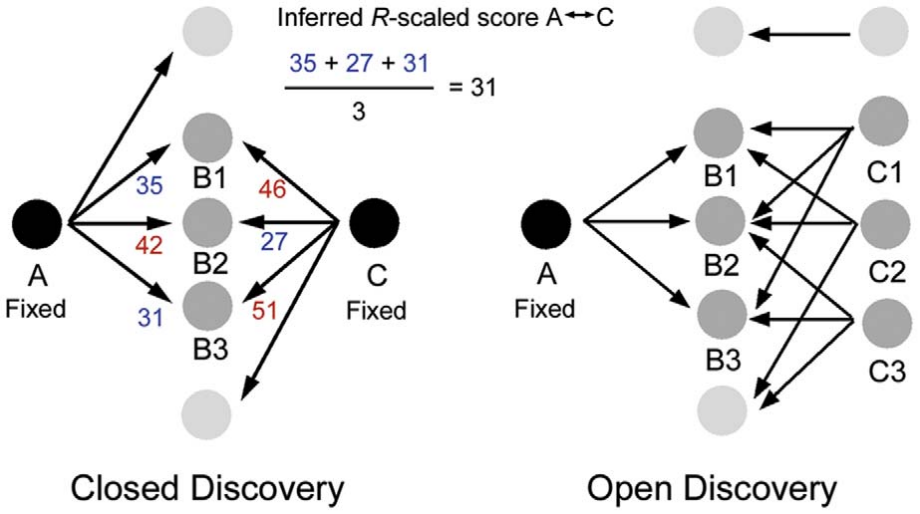
ABC-principle of hidden relationships in literature. Hidden relationships in literature between biomedical concepts (e.g., genes, diseases, drugs), for which A and C have no direct relationship, but are connected indirectly via B-intermediates, can be analyzed in a closed discovery or open discovery setting. The inferred *R*-scaled (*R*_i_) score between A and C, which is a measure for the strength of a hidden relationship, is calculated by summation of the *R*-scaled scores of the weakest links (i.e. lowest *R*-scaled score), divided by the number of intermediates.

A common method to establish relationships between biomedical concepts such as genes and pathways is co-occurrence [15]. This method is built on the assumption that biomedical concepts occurring in the same body of text are in some way biologically related. Co-occurrence-based methods can also be used to discover new, hidden relationships, assuming that if A and C both are connected with B, A and C might also have a relationship, even if there is no published relationship between A and C (Figure 1). Swanson has provided a classic example in his study in which he found that fish-oil intake is beneficial for patients suffering from Raynaud's disease, a finding that was confirmed experimentally a few years later [16], [17]. Hidden literature relationships can be used to confirm a hypothesis about a relationship between A and C in a so called closed discovery process [18]–[20]. In this process the user provides the hypothesis that A is related to C, which is then tested by mining the literature for shared biomedical concepts (B) that support the hypothesis (Figure 1). Hidden relationships can also be used to generate novel hypotheses about a relationship between A and C, in a so-called open discovery process [18], [19], [21]–[23]. In this process the user provides a starting point A (e.g. a disease) and examines the literature for hidden relationships with other biomedical concepts (C; e.g. genes, drugs) that are bridged by intermediates (B) that share co-occurrences with A and C (Figure 1).

The tools that are currently available for performing open discovery experiments are often limited to certain biomedical domains, have only limited number of keywords describing the biomedical terms, or retrieve hidden relationships formed by uninformative concepts, such as “*in vitro*” or “microarray” which are biologically less interesting [19], [22], [24]. Moreover, a bottleneck with all open discovery tools is to identify true, biologically informative, hidden relationships from spurious hits.

In a previous paper we described CoPub [25], a database of co-occurrences of ∼250.000 keywords (including gene names and symbols) in Medline abstracts. CoPub is a database in which the statistical relevance of all co-occurrences is pre-computed, which makes it possible to perform statistical analyses of the significance of the retrieved hidden relationships between biomedical concepts. In addition, CoPub contains several categories of controlled vocabularies such as genes, drugs, or diseases etc. As such, this database is ideally suited for use in the discovery of hidden relationships.

In this paper we describe CoPub Discovery, a method that uses the CoPub database for the open and closed discovery of hidden literature relationships. Statistical analysis of the results using ROC curves show that with CoPub Discovery true hidden relationships can be distinguished from true negatives. Application of this method in open ended retrieval of hidden relations yielded novel hypotheses about gene-disease, drug-disease and drug-biological process relationships which were validated bibliographically. Moreover, we used CoPub Discovery to identify two novel compounds that would interact with cell proliferation. Experimental validation showed that these compounds dose dependently inhibited T-cell proliferation.

## Results

### CoPub Discovery performance evaluation

**Figure 2:**
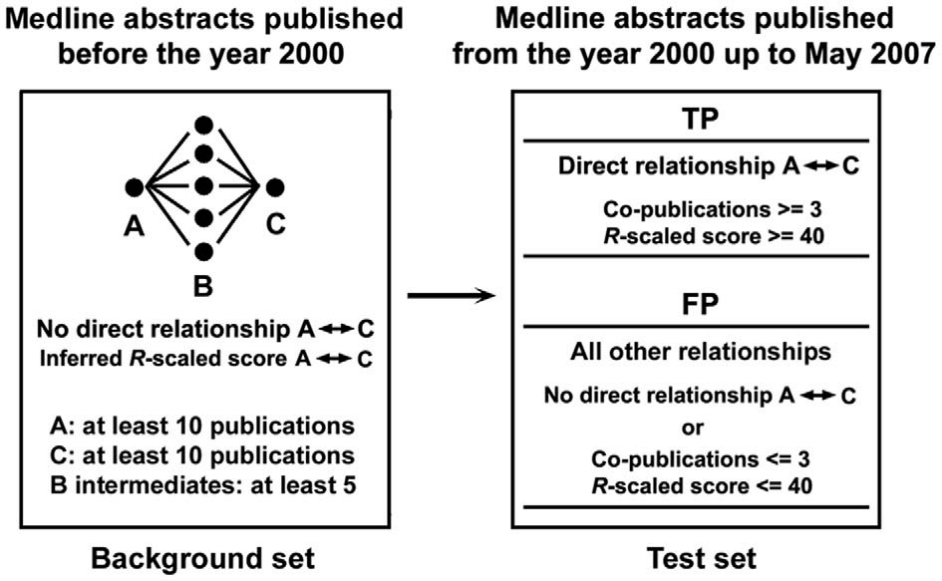
CoPub Discovery validation by literature-partitioning. A literature-partitioning analysis was performed to evaluate CoPub Discovery's ability to filter true positive (TP) from false positive (FP) hidden relationships in literature. Abstracts published in the year 2000 up to May 1, 2007 were used to validate relationships inferred from abstracts published before the year 2000.

**Figure 3:**
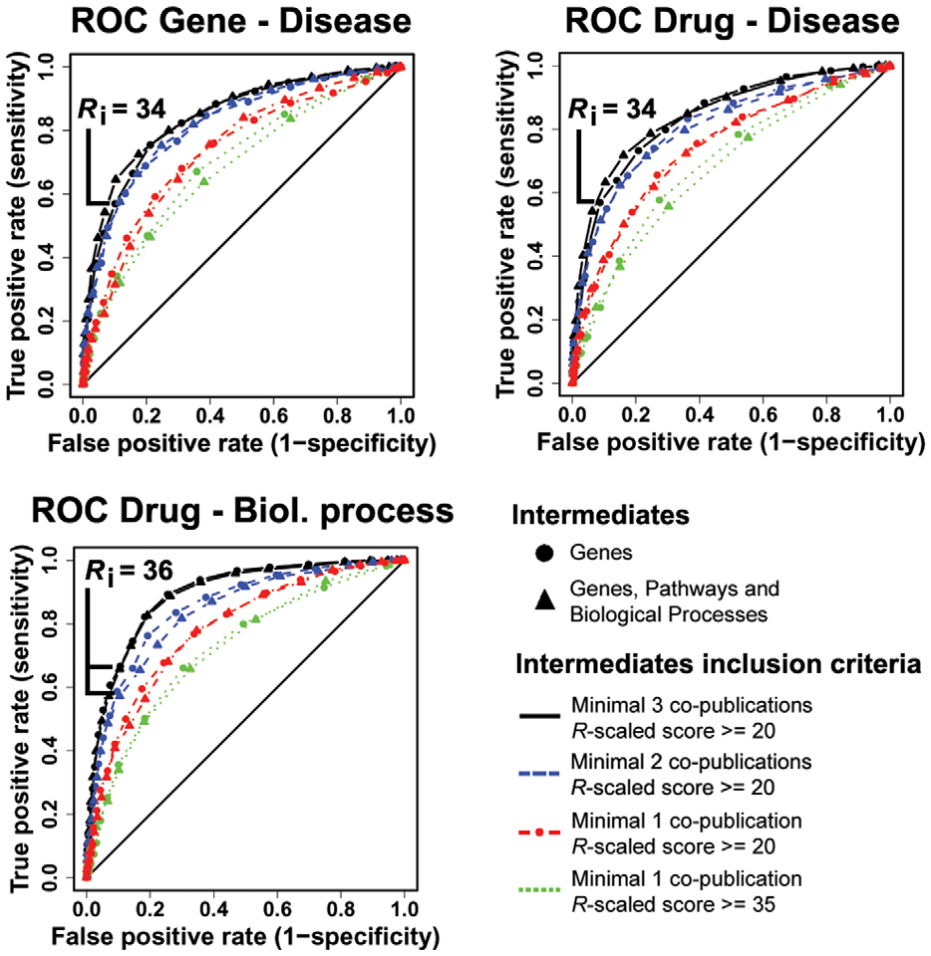
CoPub Discovery statistical evaluation. Receiver Operating Characteristic (ROC) curve analyses were performed to statistically evaluate the ability of CoPub Discovery to distinguish true positive (TP) from false positive (FP) hidden relationships in literature. In this figure, ROC curves are shown of gene-disease, drug-disease and drug-biological process hidden relationship analyses for several intermediate inclusion criteria. For each tested setting, the false positive rate was plotted against the true positive rate for each inferred *R*-scaled (*R*_i_) score cutoff.

**Figure 4:**
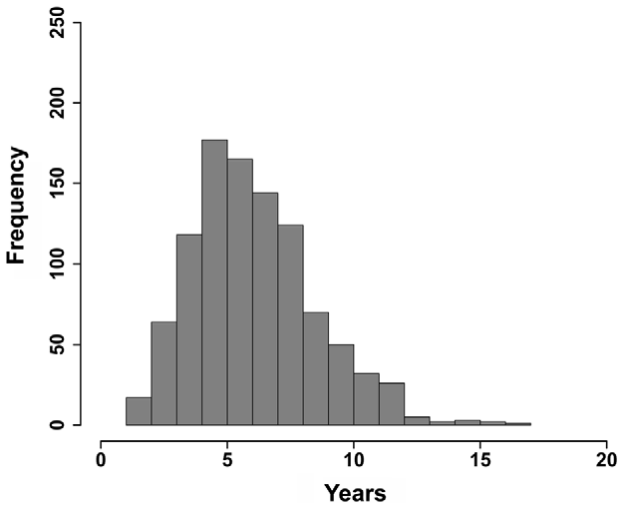
Time lag between prediction and first appearance of hidden relationships in literature. The time lag between the data needed for prediction of a hidden relationship and its actual assertion in literature is plotted for 1000 hidden relationships. For each hidden relationship, the average publication date of all A–B and B–C literature appearances was calculated and compared with the date on which A and C were first mentioned in the literature. Note: the data was derived from the literature-partitioning analysis.

As described above the challenge in the discovery of hidden relationships is to robustly discriminate true, biologically relevant, relationships (TP) from spurious, false positive hits (FP). We used Receiver Operating Characteristic (ROC) curve analyses to evaluate the ability of CoPub Discovery to distinguish TP from FP hidden relationships using literature partitioning as described in detail in the Materials and Methods section and outlined in Figure 2. All the ROC curves had AUCs (Area Under the Curve) higher than the AUC of the curve for no discrimination (Figure 3). High AUC's were obtained for all types of hidden relationships and a wide range of combinations of settings for inclusion of the intermediates. Re-running the analysis with alternative scoring schemes, such as the average instead of the minimal *R*-scaled scores between A and B, and B and C or using the average of only the 5 top scoring intermediates or using drugs rather than genes as intermediates yielded similar results (data not shown). As an additional measure of performance of CoPub Discovery, we calculated the time lag between the average publication date of all A–B and B–C intermediates and compared this date with the date of first appearance of A and C in the literature (Figure 4). The average time lag was 6.5 years, which is an indication to which extent discoveries can be accelerated when this type of hypothesis generation is used.

Taken together, these results show that CoPub Discovery is a robust method that can be applied to quickly detect a variety of biologically relevant hidden relationships.

### Case studies

After the formal validation with ROC curves we used CoPub Discovery to study a number of cases in an open discovery approach. We now used all Medline abstracts published before May 1, 2007 to find hidden relationships between genes, pathways, drugs and diseases. For each hidden relationship an inferred *R*-scaled score (*R*i) was calculated. The biological rationale of the hidden relations with the highest *R*i were studied in more detail using the underlying literature that describes the connecting intermediates, as provided in the CoPub Discovery output.

#### Case study 1: Disease-gene hidden relationships

Graves' disease (GD) is a complex autoimmune thyroid disorder, which is characterized by hyperthyroidism (i.e. over-production of thyroid hormones). Auto-antibodies against the thyroid-stimulating hormone receptor were shown to be responsible for the hyperthyroidism in GD [26]. The mechanisms behind the onset of GD are not completely understood, but it is thought that the development of GD depends on complex interactions among environmental and genetic factors [27].

CoPub Discovery was used to identify genes that might play a role in GD, but with no known relationship with GD. The analysis was conducted allowing only genes as intermediates. Several genes were found in literature with a hidden relationship with GD that had a *R*i score above the significance cutoff (Table 1a). At the top of the gene list, connected with 21 genes to GD, is *Programmed cell death 1* (PDCD1).

**Table 1:** Prediction of novel relationships between biomedical concepts using the open discovery setting.

a) Graves' Disease – Gene Hidden Relationships		
Gene	Intermediates	*R*i
Programmed cell death 1 (PDCD1)	21	34.3
CD74 molecule, major histocompatibility complex class II (CD74)	20	35.4
TAP binding protein (TAPBP)	19	34.4
CD8b molecule (CD8B)	18	34.6
CD84 molecule (CD84)	17	34.4

b) Milnacipran – Disease Hidden Relationships		
Disease/disorder	Intermediates	*R*i
Obsessive-Compulsive Disorder	7	37.3
Serotonin Syndrome	5	45.6
Serotonin Syndrome	5	45.6
Drug withdrawal symptoms	5	36.8
Tardive Dyskinesia	5	36.2
Attention Deficit Hyperactivity Disorder	5	35.4

c) Pitavastatin - Biological Process Hidden Relationships		
Biological process	Intermediates	*R*i
Monocyte Activation	26	36.0
Endothelial Cell Differentiation	25	37.2
Osteoblast Differentiation	22	36.6
Adipocyte Differentiation	22	36.0
Intracellular signalling Cascade	19	37.1

The following intermediate inclusion criteria were used to calculate the hidden relationships: minimal number of co-publications between keywords: at least 3; minimal *R*-scaled score between keywords: at least 20; intermediates used: genes (A and B), and genes, pathways and biological processes (C); literature: till May 1, 2007. The significance cutoff of *R*_i_ scores were set to a maximum false-positive rate of 0.1, which was set to 34 for disease-gene and drug-disease relationships, and set to 36 for drug-biological processes hidden relationships, as calculated in the literature partitioning analysis (Figure 2).

**Figure 5:**
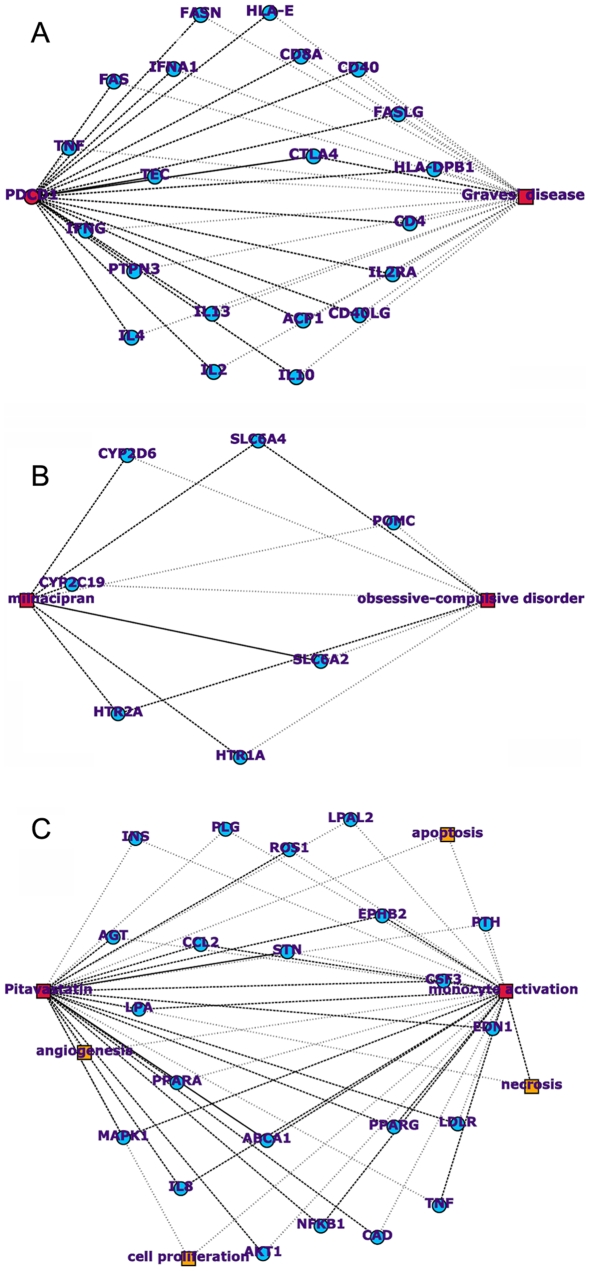
Novel predicted relationships. Hidden relationships are visualized between a) Graves' disease and *Programmed cell death 1* (PDCD1), b) milnacipran and obsessive-compulsive disorder, and c) pitavastatin and monocyte activation. A and C biomedical concepts are represented as red circles (genes) or red squares (disease, drug or biological process), whereas B-intermediates are represented as blue circles (genes) and orange squares (pathways and biological processes). The edges between nodes represent co-publications in Medline abstracts.

PDCD1 is a cell surface receptor that regulates T-cell proliferation and activation, which was linked in earlier studies to autoimmune diseases like type 1 diabetes and rheumatoid arthritis [28], [29]. Genetic studies, published after May 1, 2007 (which was not used for constructing the relationship between PDCD1 and GD) showed that small genetic effects within PDCD1 contribute to the development of GD [30], [31], and confirms that the proposed association between PDCD1 and GD by CoPub Discovery is indeed biologically relevant. One of the genes that was identified as an intermediate between PDCD1 and GD is *Cytotoxic T lymphocyte-associated antigen 4* (CTLA4) (Figure 5a). CTLA4, like PDCD1, is a negative regulator of T-cell activation [32], and polymorphisms in this gene are associated with the onset of GD [33], [34]. Studies report that CTLA4 and PDCD1 act as co-inhibitors of T-cell proliferation and activation [35], [36]. This functional association between PDCD1 and CTLA4 explains the relationship between PDCD1 and GD, and indicates that the CoPub Discovery predicted association between PDCD1 and GD was correctly identified based on biological knowledge.

#### Case study 2: Drug-disease hidden relationships

Milnacipran, a serotonin and noradrenalin reuptake inhibitor (SNRI), is a regularly prescribed drug to treat depression [37]. SNRIs prevent the reuptake of serotonin and noradrenalin by pre-synaptic cells, and thereby increase the extracellular availability of serotonin and noradrenalin to bind to post-synaptic receptors, which enhances their biological effect [38].

Depression is often accompanied by chronic pain. Therefore, antidepressants like milnacipran also become more widely applied to treat chronic pain [39], [40]. Serotonin and noradrenalin act as key mediators in various biological processes, and therefore SNRIs, due to their dual action of preventing reuptake of both serotonin and noradrenalin, are used to treat a range of distinct disorders.

We used CoPub Discovery to predict new applications for milnacipran. Several disorders were found by CoPub Discovery that had a significant, hidden relationship in literature with milnacipran using genes as intermediates (Table 1b). The top scoring disorder, connected with 7 gene intermediates, is obsessive compulsive disorder (OCD).

OCD is a common chronic anxiety disorder that can have disabling effects on both adults and children. OCD is characterized by recurrent obsessions and uncontrolled compulsions such as repetitive behavioral or mental acts that are performed in response to an obsession [41]. Marble-burying behavior in mice is recognized as a model for OCD [42]. In this model, inhibition of marble burying is correlated with reduction of anxiety, which can be achieved by treatment with selective serotonin reuptake inhibitors (SSRIs) [43]. Several studies have shown that in addition to SSRIs, SNRIs are also promising drug candidates for treatment of OCD [44], [45]. Again, to validate the predicted association between milnacipran and OCD, we inquired the literature from May 1, 2007 until present for studies that report on a functional relationship between milnacipran and OCD. Indeed, in a study published after May 1, 2007, milnacipran was found to significantly inhibit marble-burying behavior in mice [46], which demonstrates that the inferred relationship between milnacipran and OCD by CoPub Discovery appears biologically valid.

Two genes that connect milnacipran and OCD are the norepinephrine transporter SLC6A2 and the serotonin transporter SLC6A4 (Figure 5b). Milnacipran, SLC6A2 and SLC6A4 are linked in literature by studies that report on the inhibitory effect of milnacipran on norepinephrine and serotonin uptake [47], [48], whereas susceptibility to OCD was linked in literature to polymorphisms in SLC6A4 and SLC6A2 [49], [50]. These reports underpin a functional relationship between milnacipran and OCD, and shows that the predicted relationship between milnacipran and OCD by CoPub Discovery can be well explained by the biology of the intermediates.

#### Case study 3: Drug - biological process hidden relationship

Pitavastatin is a new synthetic inhibitor of 3-hydroxy-3-methyl glutaryl coenzyme A (HMG-CoA) reductase, which was shown to be a potent cholesterol-lowering agent [51]. The short-term and long-term lipid-modifying effects of pitavastatin have already been investigated in subjects with familial hypercholesterolemia, hypertriglyceridemia and type 2 diabetes mellitus [51], and the drug has been in Phase III trials in Europe, US and Japan [52].

The primary effect of a drug on its target and on related cellular processes is in many cases well known, whereas other beneficial, pleiotropic effects are often less well understood or are not immediately clear from literature. To predict additional cellular effects of pitavastatin and to understand its mode of action, CoPub Discovery was used predict relationships between biological processes and pitavastatin using genes and biological processes as intermediates.

It appeared that the four top scoring biological processes represent cell differentiation processes (Table 1c). To assess whether pitavastatin indeed affects cell differentiation, we inspected the literature from after May 1, 2007 that report on such an association. For adipocyte differentiation, indeed a direct link between pitavastatin administration and cell differentiation was reported [53]. In this study, pitavastatin was shown to have an inhibitory effect on preadipocyte differentiation into mature adipocytes by attenuating the expression of *Peroxisome proliferator activated receptor gamma* (PPARγ), a known inducer of adipogenesis [54], [55] and one of the intermediates in the hidden relationship between pitavastatin and adipocyte differentiation. This indicates that the predicted linkage between pitavastatin and adipocyte differentiation by CoPub Discovery is very likely and merits further research.

Pivastatin is connected to monocyte activation/differentiation with 26 intermediates (Table 1c). Activation of monocytes induces monocyte migration to sites of inflammation and induces differentiation of monocytes into macrophages, dendritic cells and osteoclasts [56], [57]. Inspection of the intermediates identified, among others, *Chemokine (C-C motif) ligand 2* (CCL2, also known as MCP1) and PPARγ (Figure 5c). The literature that links monocyte activation and differentiation to CCL2 and CCL2 to pitavastatin, show that CCL2 is an inducer of monocyte activation and migration [58] and that pitavastatin attenuates gene expression of CCL2 in smooth muscle and endothelial cells [59], [60]. This raises the hypothesis that pitavastatin is able to block monocyte activation and migration by downregulation of CCL2 gene expression in CCL2-secreting cells.

Similarly, the literature shows that pitavastatin downregulates the expression of PPARγ in macrophages [61]. The expression of PPARγ is upregulated in activated monocytes/macrophages and PPARγ plays a role in induction of differentiation of macrophages into foam cells [62], raising the possibility that by downregulating PPARγ expression, pitavastatin is able to suppress activation and differentiation of monocytes into macrophages. The hypothesis that pitavastatin is linked to cell differentiation is in line with studies on simvastatin, which is also a HMG-CoA reductase inhibitor, that was shown to affect cell differentiation [63], [64].

Altogether, pitavastatin is strongly linked to cell differentiation by CoPub Discovery. In cardiovascular disease, aberrant differentiation of macrophages into foam cells leads to plaque formation at vascular endothelium cells and can cause occlusion of blood vessels [65], whereas over-abundance of adipocytes causing obesity is considered to be a major risk factor for type II diabetes [66]. Although the beneficial effect of pitavastatin on atherosclerosis and diabetes has been well recognized [51], the underlying mechanisms on how pitavastatin initiates these effects have remained elusive. Based on the results of CoPub Discovery, it might be hypothesized that pitavastatin might prevent foam cell formation by blocking monocyte differentiation via suppression of CCL2 and PPARγ expression. Furthermore, the proposed relationship between pitavastatin and adipocyte differentiation, which was confirmed in the literature [53], may explain the beneficial effect of pitavastatin on obesity and diabetes [66].

Taken together, the results of CoPub Discovery show that this tool is well-suited to predict mechanisms of drug action and to derive hypothesis about the biological pathways that are involved.

#### Case study 4: Biological process - drug hidden relationship

To experimentally test a number of predicted relationships we used CoPub Discovery to search for drugs that could interfere with cell proliferation, a process for which assays are readily available. Several drugs were found to have a significant hidden relationship when using genes as intermediates with the term ‘cell proliferation’ (Table 2). From this list, two top-scoring compounds; dephostatin, a protein tyrosine phosphatase inhibitor [67], and damnacanthal, a protein tyrosine kinase inhibitor [68], were selected to test their hypothesized association with cell proliferation in an *in vitro* cell assay.

**Table 2:** Prediction of novel links between cell proliferation and drugs using open discovery.

Drug Name	# Intermediates	*R*i
Dephostatin	22	36.8
Damnacanthal	15	37.2
Aniracetam	14	36.4
Mizolastine	12	36.2
Betaseron	12	36.2

The following intermediate inclusion criteria were used to calculate the hidden relationships: minimal number of co-publications between keywords: at least 2; minimal *R*-scaled score between keywords: at least 20; intermediates used: genes; literature: till May 1^st^ 2007. The significance cutoff of *R*_i_ scores were set to a maximum false-positive rate of 0.1, which was set to 36 for the used settings, as calculated in the literature partitioning analysis (Figure 2).

**Figure 6:**
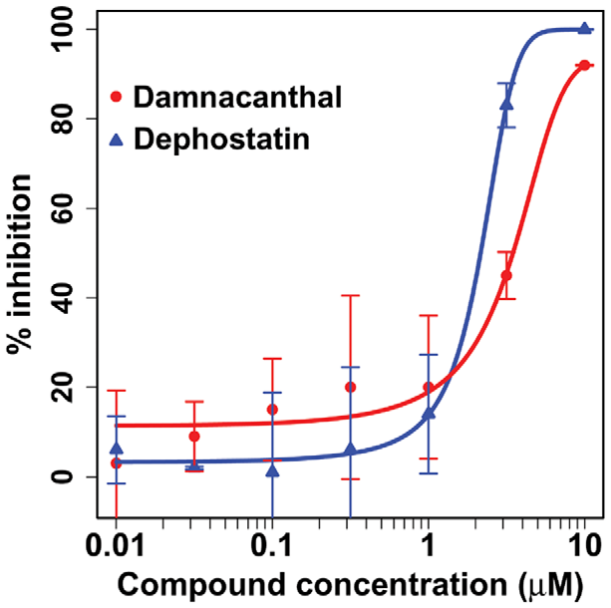
*In vitro* cell proliferation assay validates CoPub Discovery's prediction. The predicted influence of damnacanthal (red line and bullets) and dephostatin (blue line and triangles) on cell proliferation was tested in an *in vitro* cell assay. For both damnacanthal and dephostatin, the percentage of inhibition was measured and plotted against compound concentration. Both compounds were shown to inhibit cell proliferation in PBMCs when using concentrations in the 1 to 10 µM-range. The EC_50_ was estimated at 1.96 µM for dephostatin and for damnacanthal at 2.67 µM.

Human peripheral blood mononuclear cells (PBMCs) were pre-incubated with damnacanthal or dephostatin for concentrations ranging from 0.01 to 10 µM, followed by incubation with CD3/CD28 antibodies, which triggers T lymphocyte proliferation. Both damnacanthal and dephostatin inhibited proliferation of PBMCs for concentrations in the µM-range, with EC_50_'s of 2.67 µM and 1.96 µM respectively (Figure 6). Cell viability and apoptosis were not affected by damnacanthal and dephostatin indicating that damnacanthal and dephostatin specifically inhibit cell proliferation.

An earlier study showed that damnacanthal inhibits Ras function [69], which provides a mechanism of action of how damnacanthal might influence cell proliferation, as Ras oncogenes are involved in cell cycle regulation. A study performed prior May 2007 showed that dephostatin inhibits the growth of Jurkat cells [70], therefore it can be argued that the inhibitory effect of dephostatin on cell proliferation was already known. However the term ’cell proliferation‚ was not mentioned in the abstract of this paper and therefore CoPub Discovery qualified the relationship between dephostatin and ’cell proliferation‚ as novel.

These experiments provide evidence that the predicted hidden relationships with CoPub Discovery of damnacanthal and dephostatin with cell proliferation were indeed correct.

## Discussion

In this paper we described CoPub Discovery, a web-based tool that mines the Medline database for novel relationships between genes, diseases, drugs and pathways. The results show that using hidden relationships, we can successfully identify novel disease-related genes, generate novel hypotheses on drug mode of action and predict novel lead compound applications.

Drug discovery is a difficult and time-consuming process. Despite the strong increase in funding of research and development the last decade, the number of drugs that reach the market each year is lagging behind [71]. Several strategies have been adopted to bridge this gap. The use of systems biology for gaining better knowledge on the mechanisms of drug action and toxicity [72]–[75] and the use of biomarkers that are predictive for a certain biological outcome [76]–[78], are widely used solutions to improve decision making. In addition, drug repositioning, which is the use of existing drugs for new applications, is another area that is gaining much attention as a means to boost drug development [79]. Several text mining solutions have been developed to assist in and speed up the above strategies.

In a recent paper, Compillos *et al.* showed how text mining of drug labels, can be used to infer whether two drugs share the same target [80]. Our study identified several novel targets for known drugs, based on a different algorithm and another text corpus. This indicates that mining of literature is an interesting and fruitful approach to identify new drug-target relations, a first step in developing drugs towards new applications.

Detailed knowledge of the mechanism of action of a drug and the biological processes that are targeted by a drug is of importance for fine tuning drugs and biomarker discovery. In an earlier study, we showed that the application of text mining on expression data from a toxicogenomics experiment yielded detailed insight in the mode of toxicity of the tested compounds [13]. With the hidden relationship algorithm presented in this paper we provide a text mining tool that is independent of gene expression data, to improve the understanding of a drug's mechanism of action and the pathways targeted by that drug.

Although CoPub Discovery is successful in identifying novel, biologically relevant relationships in literature, several improvements may be envisioned. For example, incorporation of additional evidence for true relationships between concepts from sources other than literature, such as protein-protein interaction data or gene co-expression data, could help prioritize relationships by biological relevance. Furthermore, an additional measure of confidence could come from analyzing the relationships between the intermediates that connect A and C. A highly interconnected set of intermediates could indicate/validate higher biological relevance compared to a set with few interconnections.

Co-occurrence-based text mining does not capture the type of the extracted relationships (e.g. A binds, blocks, induces B). Therefore, in the CoPub Discovery web server the results are linked to the original abstracts in which the relationships were found. This enables the scientist to read the facts to uncover the type of relationship between A and C. A good starting point for discovery would be to look for intermediate nodes (B) that have the highest *R*-scaled scores for both node A and node C, because they have the strongest link between A and C. After selecting a few of these nodes, the researcher can perform a detailed analysis on the functional association between A and C by reading the abstracts in which A and B, and B and C are mentioned. Additionally, incorporation of natural language processing in hidden relationship analysis could assist in determining the type and direction of the relationship between A and C.

In the validation procedure of CoPub Discovery using ROC curve analysis we define FPs as A–C relationships that are predicted in the literature before the year 2000 that were not detected in subsequent literature. It might be well true that a FP is in fact a novel discovery, but is not yet discovered in subsequent literature. Furthermore, one can argue that a high area under the curve (AUC) score indicates that CoPub Discovery discovers very little that would not have been eventually discovered without it. In this respect, the 6.5 year time lag between the CoPub Discovery and the report in literature may be more indicative of the true value of CoPub Discovery; it significantly speeds up hypothesis generation, filtering and testing as was demonstrated in case example 4 in which we exactly followed this approach.

Evaluating the ROC curves in light of the performance of other text mining tools is hampered by the fact that not all of the tools are accessible or work on different text corpora or use different thesauri. Development of tools for discovery of hidden relationships would benefit from the use of expert-curated test and training sets on well-defined literature corpora, as is done in the BioCreative text mining challenges.

The statistical underpinning of CoPub Discovery provides a significant advantage over existing text mining tools applied in the area of drug development [19], [22], [24]. It allows confidence level calculations for hidden relationships and facilitates the discrimination of biologically relevant from biologically less interesting hidden relationships. To ensure the quality of the hidden relationships, several stringencies were placed on the biomedical concepts used in CoPub Discovery. For example, the biomedical concepts used in literature mining were all pre-tested for false positive generation upon inclusion in one of the biomedical concept thesauri. Furthermore, only genes and biological processes are allowed as intermediates, which avoid relationships being formed by non-informative concepts, such as ’protein‚, ’cell assay‚, etc.

In short, the results in this paper show that CoPub Discovery is able to identify novel associations between genes, drugs, pathways and diseases that have a high probability of being biologically valid. The fact that this is done rapidly, in an automated way, makes the tool especially useful in areas where large amounts of data need to be analyzed. A typical use of this tool could be to quickly rank potential new biomarkers obtained from e.g. a microarray experiment, based on their relation to diseases and drugs. CoPub Discovery could also help in drug repositioning in which list of drugs are clustered and ranked on basis of their relation with diseases and biological processes of interest.

## Materials and Methods

### Text mining Medline abstracts

Six thesauri containing human genes, Gene Ontology biological processes, liver pathologies, diseases, pathways and drugs were used to search Medline XML files containing title, abstract and substances (1966 – August 2009, http://www.nlm.nih.gov/bsd/licensee/2009_stats/baseline_doc.html), as described previously [13], [25]. The keyword thesauri are based on biological items, which represent an instance of a biological concept (e.g., a gene, a pathway), and may contain multiple keywords (e.g., a gene is assigned a full gene name, a gene symbol and gene aliases). Typical full gene names contain commas and often additional descriptions in parenthesis, which makes a full gene name an inadequate direct search term. Therefore, full gene names were processed by deleting all terms included in parentheses and allow a white space for each comma in the full name. Two-letter gene symbols and aliases were removed from the thesaurus and all other gene symbols were compared to an English dictionary to remove common English words (such as “AND”, “CELL”, etc.).

Regular expressions were used to search the compiled Medline text files for the presence of all keywords (∼250.000) from the biological concept thesauri. For the full gene name descriptions the characters "] [.-)(,:;" and space were allowed preceding and following the full gene name and also an optional “s” was permitted to follow the full gene name. Any white space in the full gene name was allowed to be a white space or a dash. The same regular expressions were applied to the non-gene biomedical concept descriptions (e.g. diseases, biological processes).

Keywords that generated a hit in a Medline abstract were stored, together with the PubMed identifiers (IDs) of the Medline records in which the hit occurred. For every biological item the hits were made non-redundant (note: multiple keywords of a biological item can occur in the same Medline abstract), resulting in a PubMed ID-biological item list. Gene symbol hits were examined for ambiguity. This was done by matching words of the full gene name in the abstract in which the gene symbol had a hit. When parts of the full gene name matched in the abstract, the gene symbol hit was regarded as a true positive; otherwise the gene symbol hit was discarded. The performance of the thesaurus-based keyword matching algorithm including the symbol disambiguation step was evaluated by repeating the human gene normalization task of the BioCreative II contest (www.biocreative.org). CoPub reached a recall of 0.78 and a precision of 0.68, resulting in an F-measure of 0.73. Based on this F-measure, CoPub would have been ranked 11^th^ out of 21 participants [81].

Co-publication of biological items (e.g. a gene with a biological process) was retrieved from the database by matching Medline abstract occurrences. An *R*-scaled score ranging from 1–100, which describes the strength of a co-citation between two biological items given their individual frequencies of occurrence, was used to assess the significance of a co-occurrence [15]. The *R*-scaled score is based on the mutual information measure (MIM) and was calculated as S = P_AB_/P_A_*P_B_ in which P_A_ is the number of hits for biological item A divided by the total number of PubMed IDs, P_B_ is the number of hits for biological item B divided by the total number of PubMed IDs, and P_AB_ is the number of co-occurrences between biological item A and biological item B divided by the total number of PubMed IDs. The relative score R is produced as a log10 conversion of S (R = ^10^log S) and the 1–100 scaled-log-transformed relative score (*R*-scaled score) as R' = 1+99 * (R – R_min_)/(R_max_ – R_min_), where R_min_ and R_max_ are the lowest and highest R values present in the biological item co-publication list, respectively. A high *R*-scaled score indicates that if two biological concepts occur in literature they are often published together, this in contrast to a low *R*-scaled score which indicates that two biological concepts often occur separately in literature and less often together. Based on previous experiences using CoPub for the interpretation of microarray data, an *R*-scaled score of above 40 can be regarded as biologically significant.

The scoring of hidden relationships between biomedical concepts was adapted from Wren's minimal MIM (MMIM) model [82] where we used an *R*-scaled score instead of a MIM score. The strength of the hidden relationship between A and C is calculated using the *R*-scaled scores between A and B, and between B and C. This inferred *R*-scaled (*R*i) score between A and C is calculated by summation of the *R*-scaled scores over the intermediates B, taking the lowest score in each pair (AB, BC), and dividing by the number of intermediates (Figure 1).

### Bibliographic prediction and validation by literature partitioning

Medline was divided into two sets (Figure 2). One set, the background set, contained abstracts published before the year 2000. The second set, the test set, contained abstracts published from the year 2000 up to May 1, 2007. Biomedical concept pairs were formed from the background set, using the following criteria: 1) The members of the pair do not co-occur in any abstract. 2) Each member of the pair occurs in at least 10 distinct abstracts. 3) The members share at least 5 intermediates.

The test set was then used to evaluate whether the pairs from the background set had a true relationship or not. A true relationship for a pair (TP) was defined using the following criteria: 1) The pair should have at least 3 co-occurrences in the test set. 2) The *R*-scaled score for this pair should be >40. All other pairs (FP) were regarded as not having a true relationship. CoPub Discovery was then evaluated for the ability to predict TPs from the background set.

To this end an inferred *R*-scaled (*R*i) score was calculated for each pair of the background set and the performance of CoPub Discovery of separating TP from FP relationships was measured with ROC curves generated by varying the *R*i threshold. The area under the curve (AUC) of the ROC curve is equal to the probability that a method will rank a randomly chosen positive instance higher than a randomly chosen negative one [83].

To allow comparison of CoPub Discovery with other literature-based discovery tools we have made the validation data of CoPub Discovery (ROC curve analyses) available in supplementary Table S1. Furthermore, we provide a web service with which the thesauri and publication data can be downloaded. The web server and web service implementation of the method described in this paper, CoPub Discovery, is available at http://www.copub.org.

### *In vitro* cell proliferation, viability and apoptosis assays

Damnacanthal (3-Hydroxy-1-methoxyanthraquinone-2-aldehyde), Merck Biosciences, Cat.No. 251650. 3,4-Dephostatin (3,4-Dihydroxy-N-methyl-N-nitrosoaniline), Merck Biosciences, Cat.No. 263202. Cell proliferation assays were performed with human peripheral blood mononuclear cells (PBMCs), stimulated with CD3 (OKT3)/CD28 (pelicluster CD28 clone CLB-CD28/1, Sanquin, the Netherlands) antibodies at a concentration of 125ng/ml and 250 ng/ml respectively in the presence or absence of compounds. Proliferation was determined after 3 days via ^3^H-thymidin incorporation for 24 hours. Viability of PBMCs was measured by Alamar Blue cell viability assay (Molecular Probes/Invitrogen, Eugene, OR). Apoptosis of PBMCs was measured using caspase GLO 3/7 activity assay (Promega, Madison, WI).

## Supporting Information

Table S1 ROC curve data used to validate CoPub Discovery - This supplementary table contains the raw data of the ROC curve analysis to validate CoPub Discovery for Disease-Gene, Drug-Disease and Drug-Biological Process hidden relationships, for several B-node inclusion criteria. The true positive rate (TPR) was calculated as: TPR = TP/TP+FN, and the false positive rate (FPR) was calculated as: FPR = FP/FP+TN (TP = True positive, FN = False negative, FP = False positive and TN = True negative).
(0.17 MB XLS)

## References

[1] Andrade MA, Bork P (2000). Automated extraction of information in molecular biology.. FEBS Lett.

[2] Jensen LJ, Saric J, Bork P (2006). Literature mining for the biologist: from information retrieval to biological discovery.. Nat Rev Genet.

[3] Andrade MA, Valencia A (1998). Automatic extraction of keywords from scientific text: application to the knowledge domain of protein families.. Bioinformatics.

[4] Raychaudhuri S, Schutze H, Altman RB (2002). Using text analysis to identify functionally coherent gene groups.. Genome Res.

[5] Perez AJ, Perez-Iratxeta C, Bork P, Thode G, Andrade MA (2004). Gene annotation from scientific literature using mappings between keyword systems.. Bioinformatics.

[6] Homayouni R, Heinrich K, Wei L, Berry MW (2005). Gene clustering by latent semantic indexing of MEDLINE abstracts.. Bioinformatics.

[7] Daraselia N, Yuryev A, Egorov S, Novichkova S, Nikitin A (2004). Extracting human protein interactions from MEDLINE using a full-sentence parser.. Bioinformatics.

[8] Shatkay H, Edwards S, Wilbur WJ, Boguski M (2000). Genes, themes and microarrays: using information retrieval for large-scale gene analysis.. Proc Int Conf Intell Syst Mol Biol.

[9] Jenssen TK, Laegreid A, Komorowski J, Hovig E (2001). A literature network of human genes for high-throughput analysis of gene expression.. Nat Genet.

[10] Chaussabel D, Sher A (2002). Mining microarray expression data by literature profiling.. Genome Biol.

[11] Blaschke C, Oliveros JC, Valencia A (2001). Mining functional information associated with expression arrays.. Funct Integr Genomics.

[12] Raychaudhuri S, Chang JT, Imam F, Altman RB (2003). The computational analysis of scientific literature to define and recognize gene expression clusters.. Nucleic Acids Res.

[13] Frijters R, Verhoeven S, Alkema W, van Schaik R, Polman J (2007). Literature-based compound profiling: application to toxicogenomics.. Pharmacogenomics.

[14] Frijters R, Fleuren W, Toonen EJ, Tuckermann JP, Reichardt HM (2010). Prednisolone-induced differential gene expression in mouse liver carrying wild type or a dimerization-defective glucocorticoid receptor.. BMC Genomics.

[15] Alako BT, Veldhoven A, van Baal S, Jelier R, Verhoeven S (2005). CoPub Mapper: mining MEDLINE based on search term co-publication.. BMC Bioinformatics.

[16] Swanson DR (1986). Fish oil, Raynaud's syndrome, and undiscovered public knowledge.. Perspect Biol Med.

[17] DiGiacomo RA, Kremer JM, Shah DM (1989). Fish-oil dietary supplementation in patients with Raynaud's phenomenon: a double-blind, controlled, prospective study.. Am J Med.

[18] Smalheiser NR, Swanson DR (1998). Using ARROWSMITH: a computer-assisted approach to formulating and assessing scientific hypotheses.. Comput Methods Programs Biomed.

[19] Hristovski D, Peterlin B, Mitchell JA, Humphrey SM (2003). Improving literature based discovery support by genetic knowledge integration.. Stud Health Technol Inform.

[20] Swanson DR, Smalheiser NR (1997). An interactive system for finding complementary literatures: A stimulus to scientific discovery.. Artif Intell.

[21] Wren JD, Bekeredjian R, Stewart JA, Shohet RV, Garner HR (2004). Knowledge discovery by automated identification and ranking of implicit relationships.. Bioinformatics.

[22] Yetisgen-Yildiz M, Pratt W (2006). Using statistical and knowledge-based approaches for literature-based discovery.. J Biomed Inform.

[23] Jelier R, Schuemie MJ, Veldhoven A, Dorssers LC, Jenster G (2008). Anni 2.0: a multipurpose text-mining tool for the life sciences.. Genome Biol.

[24] Fuller SS, Revere D, Bugni PF, Martin GM (2004). A knowledgebase system to enhance scientific discovery: Telemakus.. Biomed Digit Libr.

[25] Frijters R, Heupers B, van Beek P, Bouwhuis M, van Schaik R (2008). CoPub: a literature-based keyword enrichment tool for microarray data analysis.. Nucleic Acids Res.

[26] Rapoport B, McLachlan SM (2007). The thyrotropin receptor in Graves' disease.. Thyroid.

[27] Weetman AP (2003). Autoimmune thyroid disease: propagation and progression.. Eur J Endocrinol.

[28] Nielsen C, Hansen D, Husby S, Jacobsen BB, Lillevang ST (2003). Association of a putative regulatory polymorphism in the PD-1 gene with susceptibility to type 1 diabetes.. Tissue Antigens.

[29] Prokunina L, Padyukov L, Bennet A, de Faire U, Wiman B (2004). Association of the PD-1.3A allele of the PDCD1 gene in patients with rheumatoid arthritis negative for rheumatoid factor and the shared epitope.. Arthritis Rheum.

[30] Newby PR, Roberts-Davies EL, Brand OJ, Heward JM, Franklyn JA (2007). Tag SNP screening of the PDCD1 gene for association with Graves' disease.. Clin Endocrinol (Oxf).

[31] Hayashi M, Kouki T, Takasu N, Sunagawa S, Komiya I (2008). Association of an A/C single nucleotide polymorphism in programmed cell death-ligand 1 gene with Graves' disease in Japanese patients.. Eur J Endocrinol.

[32] Vandenborre K, Van Gool SW, Kasran A, Ceuppens JL, Boogaerts MA (1999). Interaction of CTLA-4 (CD152) with CD80 or CD86 inhibits human T-cell activation.. Immunology.

[33] Vaidya B, Oakes EJ, Imrie H, Dickinson AJ, Perros P (2003). CTLA4 gene and Graves' disease: association of Graves' disease with the CTLA4 exon 1 and intron 1 polymorphisms, but not with the promoter polymorphism.. Clin Endocrinol (Oxf).

[34] Yung E, Cheng PS, Fok TF, Wong GW (2002). CTLA-4 gene A-G polymorphism and childhood Graves' disease.. Clin Endocrinol (Oxf).

[35] Ito T, Ueno T, Clarkson MR, Yuan X, Jurewicz MM (2005). Analysis of the role of negative T cell costimulatory pathways in CD4 and CD8 T cell-mediated alloimmune responses in vivo.. J Immunol.

[36] Olive D (2006). [Lymphocyte coreceptors].. Med Sci (Paris).

[37] Puozzo C, Panconi E, Deprez D (2002). Pharmacology and pharmacokinetics of milnacipran.. Int Clin Psychopharmacol.

[38] Briley M, Prost JF, Moret C (1996). Preclinical pharmacology of milnacipran.. Int Clin Psychopharmacol.

[39] Briley M (2004). Clinical experience with dual action antidepressants in different chronic pain syndromes.. Hum Psychopharmacol.

[40] Kamata M, Takahashi H, Naito S, Higuchi H (2004). Effectiveness of milnacipran for the treatment of chronic pain: a case series.. Clin Neuropharmacol.

[41] Goodman WK (1999). Obsessive-compulsive disorder: diagnosis and treatment.. J Clin Psychiatry.

[42] Njung'e K, Handley SL (1991). Effects of 5-HT uptake inhibitors, agonists and antagonists on the burying of harmless objects by mice; a putative test for anxiolytic agents.. Br J Pharmacol.

[43] Ichimaru Y, Egawa T, Sawa A (1995). 5-HT1A-receptor subtype mediates the effect of fluvoxamine, a selective serotonin reuptake inhibitor, on marble-burying behavior in mice.. Jpn J Pharmacol.

[44] Dell'Osso B, Nestadt G, Allen A, Hollander E (2006). Serotonin-norepinephrine reuptake inhibitors in the treatment of obsessive-compulsive disorder: A critical review.. J Clin Psychiatry.

[45] Denys D, van der Wee N, van Megen HJ, Westenberg HG (2003). A double blind comparison of venlafaxine and paroxetine in obsessive-compulsive disorder.. J Clin Psychopharmacol.

[46] Sugimoto Y, Tagawa N, Kobayashi Y, Hotta Y, Yamada J (2007). Effects of the serotonin and noradrenaline reuptake inhibitor (SNRI) milnacipran on marble burying behavior in mice.. Biol Pharm Bull.

[47] Inazu M, Takeda H, Matsumiya T (2003). Functional expression of the norepinephrine transporter in cultured rat astrocytes.. J Neurochem.

[48] Vaishnavi SN, Nemeroff CB, Plott SJ, Rao SG, Kranzler J (2004). Milnacipran: a comparative analysis of human monoamine uptake and transporter binding affinity.. Biol Psychiatry.

[49] McDougle CJ, Epperson CN, Price LH, Gelernter J (1998). Evidence for linkage disequilibrium between serotonin transporter protein gene (SLC6A4) and obsessive compulsive disorder.. Mol Psychiatry.

[50] Miguita K, Cordeiro Q, Shavitt RG, Miguel EC, Vallada H (2006). Association study between the 1287 A/G exonic polymorphism of the norepinephrine transporter (NET) gene and obsessive-compulsive disorder in a Brazilian sample.. Rev Bras Psiquiatr.

[51] Kajinami K, Takekoshi N, Saito Y (2003). Pitavastatin: efficacy and safety profiles of a novel synthetic HMG-CoA reductase inhibitor.. Cardiovasc Drug Rev.

[52] Mukhtar RY, Reid J, Reckless JP (2005). Pitavastatin.. Int J Clin Pract.

[53] Nicholson AC, Hajjar DP, Zhou X, He W, Gotto AM (2007). Anti-adipogenic action of pitavastatin occurs through the coordinate regulation of PPARgamma and Pref-1 expression.. Br J Pharmacol.

[54] Shao D, Lazar MA (1997). Peroxisome proliferator activated receptor gamma, CCAAT/enhancer-binding protein alpha, and cell cycle status regulate the commitment to adipocyte differentiation.. J Biol Chem.

[55] Tamori Y, Masugi J, Nishino N, Kasuga M (2002). Role of peroxisome proliferator-activated receptor-gamma in maintenance of the characteristics of mature 3T3-L1 adipocytes.. Diabetes.

[56] Gordon S, Taylor PR (2005). Monocyte and macrophage heterogeneity.. Nat Rev Immunol.

[57] Imhof BA, Aurrand-Lions M (2004). Adhesion mechanisms regulating the migration of monocytes.. Nat Rev Immunol.

[58] Shokawa T, Yoshizumi M, Yamamoto H, Omura S, Toyofuku M (2006). Induction of heme oxygenase-1 inhibits monocyte chemoattractant protein-1 mRNA expression in U937 cells.. J Pharmacol Sci.

[59] Kaneyuki U, Ueda S, Yamagishi S, Kato S, Fujimura T (2007). Pitavastatin inhibits lysophosphatidic acid-induced proliferation and monocyte chemoattractant protein-1 expression in aortic smooth muscle cells by suppressing Rac-1-mediated reactive oxygen species generation.. Vascul Pharmacol.

[60] Morikawa S, Takabe W, Mataki C, Kanke T, Itoh T (2002). The effect of statins on mRNA levels of genes related to inflammation, coagulation, and vascular constriction in HUVEC. Human umbilical vein endothelial cells.. J Atheroscler Thromb.

[61] Han J, Zhou X, Yokoyama T, Hajjar DP, Gotto AM (2004). Pitavastatin downregulates expression of the macrophage type B scavenger receptor, CD36.. Circulation.

[62] Pelton PD, Zhou L, Demarest KT, Burris TP (1999). PPARgamma activation induces the expression of the adipocyte fatty acid binding protein gene in human monocytes.. Biochem Biophys Res Commun.

[63] Tomiyama K, Nishio E, Watanabe Y (1999). Both wortmannin and simvastatin inhibit the adipogenesis in 3T3-L1 cells during the late phase of differentiation.. Jpn J Pharmacol.

[64] Sugiyama M, Kodama T, Konishi K, Abe K, Asami S (2000). Compactin and simvastatin, but not pravastatin, induce bone morphogenetic protein-2 in human osteosarcoma cells.. Biochem Biophys Res Commun.

[65] Tabata T, Mine S, Kawahara C, Okada Y, Tanaka Y (2003). Monocyte chemoattractant protein-1 induces scavenger receptor expression and monocyte differentiation into foam cells.. Biochem Biophys Res Commun.

[66] Hajer GR, van Haeften TW, Visseren FL (2008). Adipose tissue dysfunction in obesity, diabetes, and vascular diseases.. Eur Heart J.

[67] Kakeya H, Imoto M, Takahashi Y, Naganawa H, Takeuchi T (1993). Dephostatin, a novel protein tyrosine phosphatase inhibitor produced by Streptomyces. II. Structure determination.. J Antibiot (Tokyo).

[68] Faltynek CR, Schroeder J, Mauvais P, Miller D, Wang S (1995). Damnacanthal is a highly potent, selective inhibitor of p56lck tyrosine kinase activity.. Biochemistry.

[69] Hiramatsu T, Imoto M, Koyano T, Umezawa K (1993). Induction of normal phenotypes in ras-transformed cells by damnacanthal from Morinda citrifolia.. Cancer Lett.

[70] Imoto M, Kakeya H, Sawa T, Hayashi C, Hamada M (1993). Dephostatin, a novel protein tyrosine phosphatase inhibitor produced by Streptomyces. I. Taxonomy, isolation, and characterization.. J Antibiot (Tokyo).

[71] Kola I, Landis J (2004). Can the pharmaceutical industry reduce attrition rates?. Nat Rev Drug Discov.

[72] Fischer HP (2005). Towards quantitative biology: integration of biological information to elucidate disease pathways and to guide drug discovery.. Biotechnol Annu Rev.

[73] Kell DB (2006). Systems biology, metabolic modelling and metabolomics in drug discovery and development.. Drug Discov Today.

[74] Butcher EC, Berg EL, Kunkel EJ (2004). Systems biology in drug discovery.. Nat Biotechnol.

[75] Butcher EC (2005). Can cell systems biology rescue drug discovery?. Nat Rev Drug Discov.

[76] Kuhlmann J, Wensing G (2006). The applications of biomarkers in early clinical drug development to improve decision-making processes.. Curr Clin Pharmacol.

[77] Wagner JA (2008). Strategic approach to fit-for-purpose biomarkers in drug development.. Annu Rev Pharmacol Toxicol.

[78] Colburn WA, Lee JW (2003). Biomarkers, validation and pharmacokinetic-pharmacodynamic modelling.. Clin Pharmacokinet.

[79] Ashburn TT, Thor KB (2004). Drug repositioning: identifying and developing new uses for existing drugs.. Nat Rev Drug Discov.

[80] Campillos M, Kuhn M, Gavin AC, Jensen LJ, Bork P (2008). Drug target identification using side-effect similarity.. Science.

[81] Morgan AA, Lu Z, Wang X, Cohen AM, Fluck J (2008). Overview of BioCreative II gene normalization.. Genome Biol.

[82] Wren JD (2004). Extending the mutual information measure to rank inferred literature relationships.. BMC Bioinformatics.

[83] Fawcett T (2006). An introduction to ROC analysis.. Pattern Recognition Letters.

